# Early Diabetic Retinopathy Detection from OCT Images Using Multifractal Analysis and Multi-Layer Perceptron Classification

**DOI:** 10.3390/diagnostics15131616

**Published:** 2025-06-25

**Authors:** Ahlem Aziz, Necmi Serkan Tezel, Seydi Kaçmaz, Youcef Attallah

**Affiliations:** 1 Electrical and Electronics Engineering Department, Karabuk University, 78050 Karabuk, Türkiye; nstezel@karabuk.edu.tr; 2 Department of Electrical and Electronical Engineering, Gaziantep University, 27310 Gaziantep, Türkiye; seydikacmaz@gantep.edu.tr; 3 Department of Electronics, Faculty of Electrical Engineering, University of Science and Technology of Oran Mohamed-Boudiaf (USTOMB), Oran 31000, Algeria; youcef.attallah@univ-usto.dz

**Keywords:** Diabetic Retinopathy, early detection, Optical Coherence Tomography (OCT), multifractal analysis, machine learning, Multi-Layer Perceptron (MLP), retinal imaging, computer-aided diagnosis

## Abstract

**Background/Objectives:** Diabetic retinopathy (DR) remains one of the primary causes of preventable vision impairment worldwide, particularly among individuals with long-standing diabetes. The progressive damage of retinal microvasculature can lead to irreversible blindness if not detected and managed at an early stage. Therefore, the development of reliable, non-invasive, and automated screening tools has become increasingly vital in modern ophthalmology. With the evolution of medical imaging technologies, Optical Coherence Tomography (OCT) has emerged as a valuable modality for capturing high-resolution cross-sectional images of retinal structures. In parallel, machine learning has shown considerable promise in supporting early disease recognition by uncovering complex and often imperceptible patterns in image data. **Methods:** This study introduces a novel framework for the early detection of DR through multifractal analysis of OCT images. Multifractal features, extracted using a box-counting approach, provide quantitative descriptors that reflect the structural irregularities of retinal tissue associated with pathological changes. **Results:** A comparative evaluation of several machine learning algorithms was conducted to assess classification performance. Among them, the Multi-Layer Perceptron (MLP) achieved the highest predictive accuracy, with a score of 98.02%, along with precision, recall, and F1-score values of 98.24%, 97.80%, and 98.01%, respectively. **Conclusions:** These results highlight the strength of combining OCT imaging with multifractal geometry and deep learning methods to build robust and scalable systems for DR screening. The proposed approach could contribute significantly to improving early diagnosis, clinical decision-making, and patient outcomes in diabetic eye care.

## 1. Introduction

Diabetic retinopathy (DR), a serious blood vessel problem linked to diabetes, silently progresses and is a major cause of blindness in working-age adults worldwide. Because it often shows no early warning signs, diagnosing DR in time is a significant medical hurdle. The risk of permanent vision loss from undetected DR is a major concern for patients [1]. DR develops due to long-term high blood sugar that damages the blood vessels of the retina, potentially leading to leaks, bleeding, fatty deposits, and swelling. As it worsens, blood flow to the retina is reduced, sometimes triggering the growth of fragile new blood vessels that can cause more bleeding and scarring [2]. Therefore, finding DR in its initial stages, before lasting damage occurs, is crucial for managing the disease and preventing diabetes-related blindness.

The diagnostic approach for diabetic retinopathy is based on a variety of imaging techniques, each offering distinct insights into structural and vascular changes in the retina. Fundus photography, fluorescein angiography (FA), Optical Coherence Tomography (OCT), and Optical Coherence Tomography Angiography (OCTA) are the most commonly used modalities in clinical practice [3]. OCT has become a particularly powerful and non-invasive imaging tool that provides high-resolution cross-sectional views of the retinal architecture [4]. This modality enables clinicians to detect early microstructural changes such as retinal thickening, intraretinal fluid accumulation, and the presence of microaneurysms—characteristic features of non-proliferative diabetic retinopathy (NPDR). Unlike traditional fundus imaging, OCT allows quantification of the thicknesses of the retinal layer, offering objective criteria for monitoring disease stage and progression [5].

However, OCTA extends the capabilities of conventional OCT by visualizing the retinal microvasculature without the need for contrast agents. This technique is particularly valuable for assessing capillary non-perfusion, microaneurysms, and neovascularization [6,7]. However, OCT remains more widely adopted due to its accessibility, faster acquisition time, and ability to detect early retinal edema before vascular abnormalities become apparent. Although several imaging techniques are available for the diagnosis of diabetic retinopathy, OCT remains a cornerstone of early detection and monitoring due to its precision, safety and detailed structural evaluation of the retina [8].

The human retinal vascular network presents a complex branching structure characterized by irregularity and self-similarity, which cannot be fully captured using classical Euclidean geometry. Fractal geometry offers a more appropriate mathematical approach to describing these complex biological forms [9]. Fractal analysis has been successfully applied to quantify the global complexity of retinal blood vessels, which is particularly useful in the context of diseases like diabetic retinopathy (DR), where vascular patterns are progressively disrupted [10].

However, the retinal vasculature is not uniformly complex; its local geometric features vary across different regions. Therefore, a single fractal dimension is often insufficient to describe the spatial heterogeneity observed in pathological conditions [10]. The multifractal analysis becomes highly relevant. Fractal models can be helpful for spotting general patterns, but they often miss the finer structural details, especially in complex biological systems. Because of that, some researchers prefer using multifractal analysis. It looks at a wider range of scaling behaviors to better understand how vascular structures change in different areas [11]. In diabetic retinopathy, even slight changes in how blood vessels are arranged or spaced out can be important. Multifractal techniques help bring out these subtle shifts, which might otherwise go unnoticed as the disease gradually worsens. These alterations are reflected in the distribution of multifractal parameters such as the generalized dimensions ($D_{q}$) and the singularity spectrum (f(α)), which provide a deeper insight into the underlying pathological processes affecting the retinal microcirculation [12,13], applied to retinal images—such as those acquired by fundus photography or Optical Coherence Tomography (OCT)—multifractal descriptors can highlight early abnormalities that may not yet be clinically visible. For example, in non-proliferative diabetic retinopathy (NPDR), where microaneurysms and capillary occlusions begin to alter the vascular structure, multifractal analysis can identify deviations from normal complexity, helping early diagnosis and disease staging [14].

Compared to fundus photography, which provides two-dimensional en face images limited to surface-level vascular features, Optical Coherence Tomography (OCT) offers cross-sectional, depth-resolved imaging of the retina with micrometer-scale axial resolution. This enables precise visualization of individual retinal layers, such as the inner plexiform layer, outer nuclear layer, and photoreceptor complex—regions that undergo subtle pathological changes in the early stages of diabetic retinopathy. As these microstructural alterations may not manifest as visible fundus abnormalities, OCT allows earlier and more sensitive detection of disease onset. Furthermore, the three-dimensional anatomical information captured by OCT provides richer input for classification models, enhancing feature extraction and improving predictive accuracy. For this reason, OCT has become a preferred modality for structural analysis in retinal diagnostics, particularly for early disease detection workflows.

In recent years, the rapid development of machine learning (ML) techniques has significantly impacted the field of medical image analysis, offering powerful tools for the early detection and classification of diabetic retinopathy [15]. Unlike traditional image processing approaches, which rely heavily on handcrafted features, machine learning algorithms are capable of learning complex representations directly from imaging data. In particular, supervised learning models have been widely employed to classify retinal images into different stages of DR based on annotated datasets, while unsupervised and semi-supervised methods have shown potential in exploring unlabeled data for pattern discovery [16].

These techniques have been applied across various imaging modalities, including fundus photography, Optical Coherence Tomography (OCT), and OCT Angiography (OCTA), to detect subtle structural and vascular changes indicative of disease progression [17]. The integration of ML models has enhanced diagnostic accuracy, consistency, and efficiency, and enabled the development of automated screening systems, especially valuable in low-resource or high-demand clinical environments. Furthermore, the combination of machine learning with advanced image analysis techniques—such as fractal and multifractal analysis—has opened new perspectives for capturing both global and local features of the retinal microarchitecture [18].

Several recent studies have explored the integration of multifractal feature extraction with classification algorithms to enhance the automated detection of retinal diseases, particularly diabetic retinopathy (DR). Lei Yang et al. (2024) [19] proposed a hybrid approach combining two-dimensional empirical mode decomposition and multifractal analysis to extract detailed morphological features from retinal images. They aimed to classify diabetic retinopathy based on the complexity and variation of retinal structures. G. El Damrawi et al. (2020) [20] explored the use of multifractal geometry to analyze OCTA images to distinguish between healthy and non-proliferative DR cases and integrated a neural network classifier to support early automated diagnosis. Devanjali Relan et al. (2020) [21] applied multifractal analysis to high-resolution fundus images to evaluate its ability to distinguish between healthy, diabetic retinopathy, and glaucomatous cases. They emphasized the role of segmented vascular structures in capturing meaningful differences across subgroups. M. Rizzo et al. (2024) [13] investigated the predictive power of vascular tortuosity features in OCTA images for detecting diabetic retinopathy using machine learning algorithms. Their approach also incorporated additional vascular descriptors, including fractal and geometrical metrics, to enhance the classification performance. Tiepei Zhu et al. (2019) [22] conducted a study using multifractal analysis on projection artifact-resolved OCTA images to assess microvascular impairments across different DR stages. They demonstrated that multifractal geometric features correlated strongly with disease severity and outperformed traditional Euclidean metrics in early DR detection. Mohamed M. Abdelsalam (2020) [13] proposed an effective methodology for early DR detection using OCTA images based on blood vessel reconstruction and the extraction of key vascular features such as intercapillary areas, FAZ perimeter, and vessel density. These features were used to train an artificial neural network to classify between diabetic subjects without DR and those with mild-to-moderate NPDR. The approach demonstrated high accuracy and speed, highlighting the value of combining image enhancement with neural network classification in early DR diagnosis. In a key study, Mohamed M. Abdelsalam et al. (2021) [12]. proposed a novel framework for the early detection of non-proliferative diabetic retinopathy (NPDR) by analyzing macular OCTA images through multifractal geometry. The retinal microvascular network was characterized using multifractal parameters such as generalized dimensions and singularity spectrum, which reflect the complex, irregular vascular structures affected by early DR changes. These multifractal features served as discriminative inputs for a supervised machine learning classifier (Support Vector Machine), enabling the automatic identification of early NPDR. This approach highlighted the effectiveness of multifractal geometry in capturing subtle microvascular alterations, and its potential adaptability for recognizing other DR stages or vascular-related retinal diseases. The methodology closely aligns with our current research focus, emphasizing the value of multifractal descriptors in enhancing diagnostic precision from OCTA data.

Optical Coherence Tomography (OCT) has become an essential imaging modality in ophthalmology, particularly in the early detection and monitoring of diabetic retinopathy (DR). Its ability to provide non-invasive, high-resolution cross-sectional images of the retinal layers makes it a powerful tool for identifying subtle structural changes associated with the progression of DR, such as retinal thickening, microaneurysms, or fluid accumulation. Unlike traditional imaging techniques that primarily capture surface-level information, OCT enables clinicians and researchers to visualize the deeper microstructural alterations caused by diabetic complications [3,23].

In recent years, the integration of OCT imaging with automated analytical tools has gained growing attention—for instance, Kh. Tohidul Islam et al. (2019) [24] proposed a deep transfer learning framework to identify diabetic retinopathy from OCT images. Their approach involved retraining pre-existing deep learning models to serve as feature extractors, followed by the application of conventional classifiers for disease detection. This work highlighted the potential of combining OCT with artificial intelligence to enhance diagnostic accuracy and efficiency in DR detection. Building on the value of OCT imaging in diagnosing diabetic retinopathy, the study by M. Sakthi Sree Devi et al. (2021) [25] presents a targeted approach for automated detection of DR through detailed retinal layer analysis. The authors utilize high-resolution OCT images to segment seven distinct retinal layers using the Graph-Cut method, a powerful algorithm driven by gradient information. By extracting critical features such as retinal layer thickness and signs of neovascularization, their methodology effectively distinguishes between healthy and diabetic retinopathy subjects. This study further underscores the potential of OCT-based structural biomarkers in facilitating early and accurate DR detection through image analysis techniques. A. Sharafeldeen et al. (2021) [26] proposed a CAD system for early diabetic retinopathy detection using OCT B-scans by combining morphological features (layer thickness, tortuosity) with high-order reflectivity markers. These features were extracted from segmented retinal layers and classified using SVMs and neural network fusion, highlighting the effectiveness of integrating anatomical and reflectivity cues for accurate early diagnosis. In the same context, Mahmoud Elgafi et al. (2022) [27] introduced a robust approach for diabetic retinopathy detection based on 3D feature extraction from OCT images. Their method involves segmenting individual retinal layers, from which volumetric features such as layer-wise reflectivity and thickness are derived. These features are then processed using a backpropagation neural network for classification. This study underlines the diagnostic value of 3D structural biomarkers within OCT scans, reinforcing the role of this imaging modality in the automated early-stage identification of DR.

Our study presents a novel and comprehensive framework for the early detection of Diabetic Retinopathy using Optical Coherence Tomography (OCT) images, addressing a gap in current research, which predominantly focuses on Optical Coherence Tomography Angiography (OCTA) due to its enhanced vascular detail but limited availability. Our approach leverages a large and accessible OCT dataset, enabling broader clinical applicability. The preprocessing pipeline, developed in MATLAB R2022a, ensures consistent image quality and normalization. We then apply multifractal analysis using the box-counting method to extract meaningful textural descriptors that characterize pathological alterations in retinal structure. These descriptors are subsequently used to train and evaluate a Multi-Layer Perceptron (MLP) classifier, demonstrating robust performance. To the best of our knowledge, this is the first study to integrate multifractal analysis of OCT images with an MLP classifier for DR detection, offering a scalable and practical solution for early-stage screening.

The remainder of this paper is organized as follows: Section 2 presents the proposed methodology. Section 3 reports and discusses the experimental results, highlighting the classification performance and comparing it with other models. Finally, Section 4 concludes the study and outlines possible directions for future research.

## 2. Methodology

The proposed approach introduces a novel and effective methodology for the early detection of diabetic retinopathy (DR) based on the structural analysis of Optical Coherence Tomography (OCT) images. Unlike prior works that rely primarily on OCTA or fundus photography, our method leverages the anatomical information provided by OCT scans, which are more accessible in standard clinical settings and less affected by motion artifacts or contrast issues.

One of this study’s key innovations is applying multifractal geometry to OCT data, allowing for the quantification of complex spatial patterns and irregularities that emerge in the early stages of DR [25]. This analysis enables the detection of microstructural retinal changes that may not be easily visible through conventional imaging or manual inspection.

In addition, the use of a neural-based classification model (MLP) contributes to enhanced detection accuracy, capturing nonlinear relationships between subtle image patterns associated with disease onset. Compared to traditional imaging-based DR detection methods that often require vascular visibility (such as OCTA or fundus images), our approach provides a non-invasive, structure-based, and clinically scalable solution for early detection.

Furthermore, the methodology has been validated on a large and balanced dataset, ensuring robustness, generalizability, and suitability for deployment in real-world diagnostic workflows.

**Figure 1:**
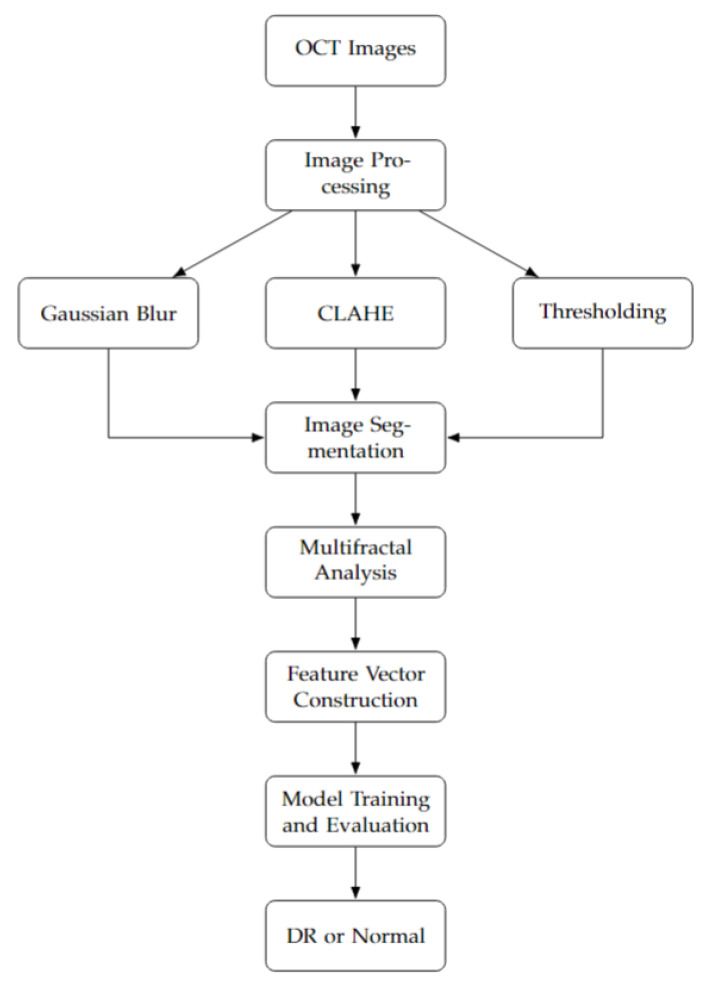
Our methodology flowchart.

The flowchart in Figure 1 outlines the full methodology of our proposed diabetic retinopathy detection system. Beginning with a balanced dataset of 6000 OCT images, we first apply several preprocessing steps, including Gaussian blur, local contrast enhancement (CLAHE), thresholding, and binarization. These steps enhance structural visibility and reduce noise, preparing the images for analysis. We then perform multifractal analysis to characterize the complex spatial structures present in the OCT scans. The resulting descriptors are compiled into a structured dataset, which undergoes normalization and K-fold cross-validation to ensure robustness and mitigate overfitting.

A range of machine learning classifiers are trained and evaluated, and the MLP model is selected for its superior performance in classifying images into DR and normal classes. This model successfully captures the nonlinear relationships within multifractal patterns, making it well-suited for early DR screening.

### 2.1. OCT Dataset

In this study, we used the publicly available Retinal OCT Image Classification—C8 dataset compiled by Obuli Sai Naren (2021) [28]. It consists of 24,000 high-resolution OCT images categorized into eight retinal conditions. The images were collected from multiple open-access sources (e.g., Kaggle, OpenICPSR), then preprocessed and class-balanced to ensure uniform representation across classes.

For the purpose of this work, we selected two categories—normal and diabetic retinopathy—to focus specifically on binary classification for early DR detection using OCT-based structural features. The multi-source composition of the dataset ensures a certain degree of pathological and imaging variability, which supports the development of generalizable models. However, detailed clinical metadata such as patient demographics is not provided. While this does not compromise the current study’s experimental scope, future validation on clinically annotated and demographically stratified datasets would help further assess the external applicability of the proposed approach in broader clinical environments.

### 2.2. OCT Image Processing

To enhance and prepare OCT images for further analysis and classification, a comprehensive preprocessing pipeline was implemented using the open-source Fiji platform (a distribution of ImageJ) through a custom-developed macro script [29]. The main goals of this pipeline were to enhance image contrast, suppress noise, and segment retinal structures accurately to isolate clinically relevant features indicative of diabetic retinopathy (DR). Preprocessing is a crucial step in medical image analysis as it improves the quality and interpretability of the input data, which in turn enhances the performance of subsequent feature extraction and classification algorithms. Each image in the dataset underwent the following sequence of processing operations:

#### 2.2.1. Gaussian Blurring

To suppress high-frequency noise while retaining important structural details, a Gaussian blur was applied with a standard deviation (σ) of 2. The two-dimensional Gaussian function used for convolution is defined as:

(1) $$G\left(x,y\right)=\frac{1}{2\pi \sigma^{2}}\;\exp\left(-\frac{x^{2}+y^{2}}{2\sigma^{2}}\right)$$

This low-pass filter smooths pixel intensity variations and aids in reducing noise and small-scale artifacts that could interfere with segmentation [30].

#### 2.2.2. Contrast Enhancement

Local contrast enhancement was performed using the CLAHE (Contrast-Limited Adaptive Histogram Equalization) algorithm. Unlike traditional histogram equalization, CLAHE operates on small contextual regions (tiles) of the image and redistributes pixel intensities to enhance local contrast while limiting amplification via a predefined clip limit. This technique is especially effective for medical images in which subtle differences in tissue structures are diagnostically significant [31]. CLAHE was configured with a block size of 127, histogram bins of 256, and a clip limit of 3, which enhanced the visibility of retinal layers and vascular abnormalities in darker image regions [32]. The entire pipeline was implemented as a Fiji macro, which ensured batch processing of large image sets while maintaining consistency and reproducibility of the preprocessing steps (Algorithm 1) [33].
**Algorithm 1** Image preprocessing with Gaussian blur and CLAHE.  1:Prompt the user to select the input folder containing the images.  2:Prompt the user to select the output folder for processed images.  3:List all files in the input folder.  4:**for** each file in the list **do**  5:    **if** file extension is .png, .tif, or .jpg **then**  6:      Open the image.  7:      Apply Gaussian Blur with σ=2.  8:     Apply CLAHE with block size 127, histogram bins 256, and maximum slope 3.  9:      Generate a new filename by adding _processed before the extension.10:      Save the processed image as PNG in the output folder.11:      Close the image.12:  **end if**13:**end for**

### 2.3. Multifractal Analysis

Multifractal analysis is a powerful mathematical framework used to characterize complex patterns and textures that exhibit scale-invariant properties. In the context of medical imaging, particularly OCT images, multifractal analysis allows for the quantification of structural heterogeneity and irregularity in retinal tissues, which may correlate with pathological changes due to diabetic retinopathy. To perform this analysis, we used the Multifrac v1.0.0 plugin—an extension developed for the ImageJ platform—which supports the multifractal characterization of 2D and 3D image data. The plugin is freely available and can be accessed via the official ImageJ v2 website (https://imagej.net/ij/index.html, accessed on 27 December 2024). Multifrac facilitates the computation of multifractal parameters using the box-counting algorithm, a widely adopted approach for fractal dimension estimation. This tool was chosen due to its reliability, ease of integration with ImageJ, and its ability to handle grayscale images with options for preprocessing such as resizing, binarization, and scale restriction. It provides automated analysis and saves results along with processed images into structured directories. For more technical details, readers are referred to the original documentation and publication by Torre et al. (2020) [34].

In this study, we applied 2D multifractal analysis on our preprocessed OCT images using the default settings of the plugin, focusing on the white pixels after binarization to emphasize retinal structures of interest. The multifractal analysis yields a set of features that provide insight into the spatial complexity and scaling behavior of the image textures The primary descriptors extracted through this process are as follows:

- **Generalized dimensions**:
  − Box-counting dimension ($D_{B}$).
  − Information dimension ($D_{I}$).
  − Correlation dimension ($D_{C}$).
- **Singularity spectrum**: Characterizes the distribution of singularities (local scaling exponents) in the image and captures its multifractal nature.

These descriptors form the feature vector used in subsequent classification stages. In the following subsections, we will present the mathematical foundations and interpretation of these multifractal features.

### 2.4. Multifractal Features Extraction

Multifractal analysis provides a rich description of complex structures by characterizing the spatial heterogeneity of singularities in an image. In our study, two types of features are extracted from each OCT image: the generalized dimensions and the singularity spectrum.

#### 2.4.1. Generalized Dimensions

The generalized dimensions $D_{q}$ characterize how measures (such as pixel intensity distributions) scale across different orders *q* [35]. They are defined by the formula

(2) $$D_{q}=\frac{1}{q-1}\lim_{\epsilon \to 0}\frac{\log\sum_{i}p_{i}^{q}\left(\epsilon \right)}{\log\epsilon }$$

where

- ϵ is the box size;
- $p_{i}\left(\epsilon \right)$ is the probability measure in the *i*-th box.

**Figure 2:**
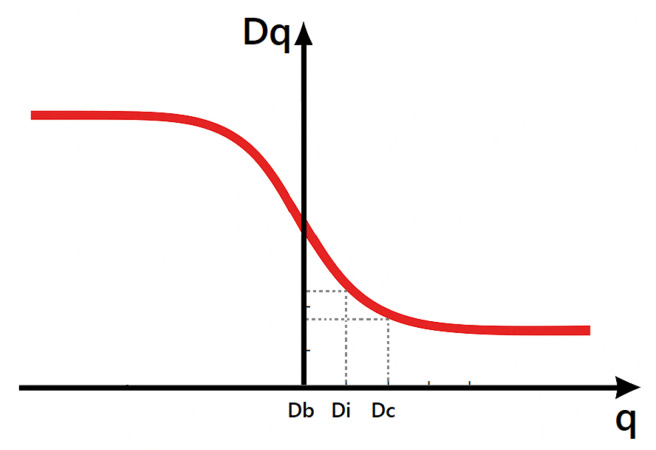
Generalized dimension curve: $D_{B}$, $D_{I}$, and $D_{C}$ extracted at q=0,1,2 respectively.

Different generalized dimensions highlight different aspects of the image structure:

- **Box-counting dimension** ($D_{B}$) for (q=0):
  (3) $$D_{B}=\lim_{\epsilon \to 0}\frac{\log N(\epsilon )}{\log(1/\epsilon )}$$
  where N(ϵ) is the number of non-empty boxes. $D_{B}$ measures the global geometric complexity of the OCT image. Higher complexity may indicate microstructural alterations such as exudates, microaneurysms, or early neovascularization characteristic of proliferative stages of DR.
- **Information dimension** ($D_{I}$) for (q=1):
  (4) $$D_{I}=\lim_{\epsilon \to 0}\frac{\sum_{i}p_{i}\left(\epsilon \right)\log p_{i}\left(\epsilon \right)}{\log\epsilon }$$
  $D_{I}$ reflects the heterogeneity in the distribution of intensity values. Increased irregularity in DR lesions (e.g., localized edema or deposits) leads to a higher entropy captured by $D_{I}$.
- **Correlation dimension** ($D_{C}$) for (q=2):
  (5) $$D_{C}=\lim_{\epsilon \to 0}\frac{\log\sum_{i}p_{i}^{2}\left(\epsilon \right)}{\log\epsilon }$$
  $D_{C}$ emphasizes clustering patterns. In DR-affected regions, pixel intensities often show strong local correlations due to lesions and structural distortions in retinal layers, making $D_{C}$ a sensitive descriptor for disease severity (Figure 2).

#### 2.4.2. Singularity Spectrum

The singularity spectrum f(α) provides a deeper characterization of the distribution of singularities. It describes how different singularity strengths are distributed spatially within the image [36]. The relationship between *q*, α, and f(α) is

(6) $$\begin{array}{cc} \alpha (q) & =\frac{d}{dq}\left[\left(q-1\right)D_{q}\right] \end{array}$$

(7) $$\begin{array}{cc} f(\alpha ) & =q\alpha -\left(q-1\right)D_{q} \end{array}$$

where

- α is the Hölder exponent (local regularity).
- f(α) is the fractal dimension of the set of points with singularity strength α.
- $\alpha_{\min}$: Minimum singularity strength—indicates the most irregular structures (e.g., sharp edges or abrupt intensity changes from hemorrhages or exudates).
- $\alpha_{\max}$: Maximum singularity strength—reflects the smoothest regions (healthy retinal layers).
- $\alpha_{\mathrm{center}}$: Value of α corresponding to the maximum f(α)—represents the most dominant singularity type.
- $f{\left(\alpha \right)}_{\max}$: Maximum height of the singularity spectrum—related to the richness or abundance of dominant structures.
- Spectrum width: $\alpha_{\max}-\alpha_{\min}$—quantifies the degree of multifractality; wider spectra suggest greater textural diversity, typical in advanced DR stages.
- Symmetric shift: $\alpha_{\mathrm{center}}-\frac{\alpha_{\min}+\alpha_{\max}}{2}$—measures the asymmetry; positive or negative shifts may indicate prevalence of finer (positive shift) or coarser (negative shift) textures, respectively.

**Figure 3:**
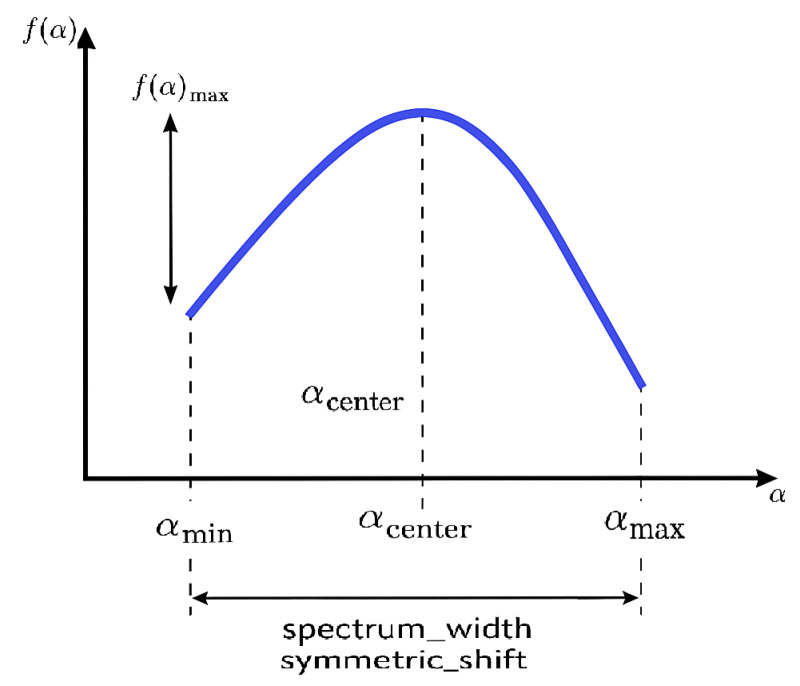
Singularity spectrum: illustrating $\alpha_{\min}$, $\alpha_{\max}$, $\alpha_{\mathrm{center}}$, $f{\left(\alpha \right)}_{\max}$, spectrum width, and symmetric shift.

Variations in singularity spectrum parameters, specifically $\alpha_{\min}$, $\alpha_{\max}$, and the spectrum width $\Delta \alpha =\alpha_{\max}-\alpha_{\min}$, serve as sensitive indicators of structural alterations within the retinal layers induced by diabetic retinopathy (DR) [12,19] (Figure 3).

The minimum singularity exponent $\alpha_{\min}$ is typically associated with regions of high-intensity concentration, corresponding to densely active pathological zones such as microaneurysms or neovascular tufts. Conversely, $\alpha_{\max}$ reflects sparsely distributed, low-density regions, which may be indicative of vessel dropout or retinal thinning [12,19].

As DR progresses from early non-proliferative stages to more advanced forms, spatial heterogeneity within the retinal microvasculature increases. This progression is quantitatively reflected in the broadening of the singularity spectrum, observed as an increase in the width Δα, suggesting a greater diversity of local scaling behaviors. In early DR, only subtle shifts in $\alpha_{\min}$ and $\alpha_{\max}$ are typically observed, corresponding to microstructural changes that may remain undetectable in conventional clinical imaging.

In contrast, moderate to severe DR stages often exhibit a markedly asymmetric singularity spectrum, highlighting uneven pathological alterations across different retinal regions [19].

Thus, multifractal parameters do not merely quantify geometric complexity; they encapsulate clinically significant information regarding tissue integrity. A broader and more asymmetric singularity spectrum is strongly associated with increased structural irregularity and disease progression. Consequently, these descriptors provide a robust quantitative framework for the early diagnosis, staging, and monitoring of diabetic retinopathy based on OCT image analysis [23].

Table 1 summarizes the multifractal features extracted from OCT images and highlights their clinical relevance for diabetic retinopathy (DR) detection. These metrics provide quantitative insights into microstructural degradation, textural irregularities, and pathological variability in retinal tissues, serving as valuable biomarkers to differentiate between healthy and diabetic-affected retinas.

#### 2.4.3. Case Study: Multifractal Analysis of DR and Normal OCT Images (Index 141)

**Figure 4:**
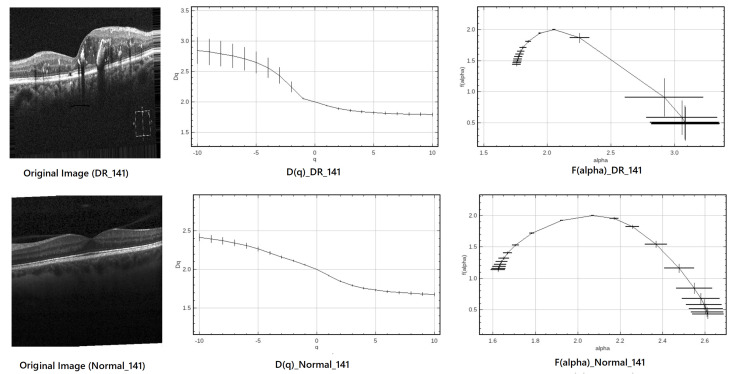
Multifractal analysis of DR_141 (**top**) and Normal_141 (**bottom**). Left: original OCT image; center: D(q) curve; right: f(α) singularity spectrum.

The discriminatory capability of multifractal analysis in retinal OCT images is illustrated through two representative case studies: one pathological case (DR_141) corresponding to a patient with diabetic retinopathy, and one healthy control (Normal_141). For each case, the original OCT scan, the computed generalized dimension curve D(q), and the singularity spectrum f(α) are presented in Figure 4. These examples clearly highlight the differences in multifractal properties between healthy and pathological retinal tissues.

The multifractal descriptors extracted from both images are summarized in Table 2, showing clear differences between the pathological and healthy samples.

As presented in Table 2, the multifractal descriptors extracted from a representative diabetic retinopathy case (DR_141) and healthy control (Normal_141) exhibit relatively close numerical values for specific parameters, such as the box-counting dimension ($D_{B}$) and the maximum of the multifractal spectrum ($f{\left(\alpha \right)}_{\max}$). At first glance, this apparent similarity might suggest a limited discriminative capacity when considering individual descriptors in isolation. However, such observations must be interpreted within the context of a higher-dimensional feature space, where even slight variations across multiple parameters can contribute to meaningful class differentiation.

Indeed, multifractal features are inherently interrelated, and their diagnostic relevance often emerges not from isolated values but from the way they interact and co-vary with one another. For instance, differences in $\alpha_{\min}$, $\alpha_{\max}$, the spectrum width (Δα), and the symmetry shift reveal nuanced changes in the local singularity structure of the retinal texture. These subtle alterations, although not visually striking in a univariate comparison, may indicate underlying pathological changes.

To leverage the full potential of these interdependencies, a machine learning classifier is introduced. Unlike traditional threshold-based methods, this classifier can model complex, nonlinear relationships across all features simultaneously. This allows it to capture latent patterns and interactions that are not evident through direct inspection of individual descriptors, thereby providing a more comprehensive and robust framework for distinguishing between normal and pathological retinal images.

From the D(q) curves, both diabetic retinopathy (DR) and healthy retinal images exhibit a decreasing trend with increasing *q*, confirming their multifractal nature. The steeper decline observed in DR cases indicates greater spatial irregularity and structural disruption, which are hallmark characteristics of diabetic retinopathy. This difference is further highlighted by the f(α) spectrum. The DR spectrum appears broader and more asymmetric, quantified by

(8) $$\Delta \alpha =\alpha_{\max}-\alpha_{\min},$$

The left-skewness of the DR spectrum suggests a dominance of coarse singularities—areas of low local regularity—typically associated with pathological changes such as hemorrhages or neovascularization. In contrast, the healthy retina presents a narrower and more symmetric spectrum, reflecting a more regular and homogeneous retinal architecture.

Overall, these findings underscore the discriminative power of multifractal descriptors—especially spectrum width and asymmetry—in differentiating retinal abnormalities. This supports their potential utility in the early detection and screening of diabetic retinopathy.

### 2.5. Multi-Layer Perceptron (MLP)

In this study, the multifractal features extracted from OCT images—such as the box-counting dimension, information dimension, and correlation dimension—were used as input to a Multi-Layer Perceptron (MLP) for the classification of diabetic retinopathy. The MLP is a supervised learning model composed of an input layer, multiple hidden layers, and an output layer, where each neuron in a layer is connected to neurons in the next via weighted connections and biases [37].

We employed the MLP classifier from the scikit-learn library with the following configuration: two hidden layers consisting of 128 and 64 neurons, respectively, a ReLU activation function, an Adam optimizer, and a maximum of 1000 iterations for training. The network architecture is designed to learn complex, nonlinear mappings from the multifractal feature space to the binary output labels (healthy or diabetic retinopathy).

The activation of neurons in each hidden layer is governed by the Rectified Linear Unit (ReLU) function, defined as

(9) $$ReLU\left(x\right)=\left\{\begin{array}{cc} x & \mathrm{if}\;x>0\\ 0 & \mathrm{otherwise} \end{array}\right.$$

The forward pass of the network for a given hidden layer *h* is expressed as

(10) $$a^{\left(h\right)}=ReLU\left(W^{\left(h\right)}\cdot a^{\left(h-1\right)}+b^{\left(h\right)}\right)$$

where $a^{\left(h\right)}$ denotes the activation output at the *h*-th hidden layer, $W^{\left(h\right)}$ is the weight matrix, and $b^{\left(h\right)}$ is the bias vector. The final output layer produces a probability score used for classification, using a logistic sigmoid function when binary classification is required:

(11) $$y=\sigma (W^{\left(out\right)}\cdot a^{\left(L\right)}+b^{\left(out\right)})$$

where $a^{\left(L\right)}$ represents the activations from the last hidden layer, and $\sigma \left(x\right)=\frac{1}{1+e^{-x}}$ is the sigmoid function.

The use of the Adam optimizer allows for adaptive learning rates, contributing to faster and more stable convergence during training. This MLP configuration proved effective in learning discriminative patterns from the multifractal features, facilitating accurate classification between healthy and diabetic retinopathy cases.

## 3. Results and Discussion

### 3.1. Hyperparameter Optimization and Experimental Setup

In order to ensure a fair and robust comparison across all classifiers, extensive hyperparameter tuning was conducted. The hyperparameters were selected through a combination of empirical testing, grid search, and literature-guided heuristics. Multiple experiments were carried out for each model to identify the configuration that yields the best trade-off between accuracy and generalization performance. The final optimized values were chosen based on their consistent performance across different trials.

Table 3 summarizes the hyperparameters selected for each classifier. These configurations were fixed across all experiments to maintain consistency and comparability between models. The hyperparameters presented above were fixed for all subsequent evaluations in order to preserve the integrity of the comparisons. Special care was taken to prevent overfitting or underfitting, with regularization parameters and model complexities adjusted accordingly.

All experiments were conducted on the Kaggle platform using a P100 GPU runtime environment to accelerate training, especially for the deep learning-based model (MLP Neural Network). A stratified K-fold cross-validation strategy (with k=5) was employed to enhance the reliability of the results. For each fold, the dataset was split into 80% for training and 20% for testing, ensuring that the distribution of the classes remained consistent across all splits. This consistent experimental design across all models ensures that performance comparisons are both fair and statistically meaningful.

### 3.2. Evaluation Metrics

To objectively assess the performance of the classification models, a comprehensive set of evaluation metrics was utilized. These metrics offer insights into different aspects of model performance, particularly in medical imaging where both false positives and false negatives carry significant implications. The following metrics were considered:

- **Accuracy**: Represents the proportion of correctly predicted instances (both positive and negative) over the total number of instances. It is defined as
  (12) $$\mathrm{Accuracy}=\frac{TP+TN}{TP+TN+FP+FN}$$
  where TP = true positives; TN = true negatives; FP = false positives; and FN = false negatives.
- **Precision**: Measures the ability of the classifier to return only relevant instances among the predicted positives. High precision indicates a low false positive rate:
  (13) $$\mathrm{Precision}=\frac{TP}{TP+FP}$$
- **Recall (Sensitivity)**: Represents the model’s ability to correctly identify all relevant instances among the actual positives. It is also known as **Sensitivity**:
  (14) $$\mathrm{Recall}=\mathrm{Sensitivity}=\frac{TP}{TP+FN}$$
- **F1-Score**: The harmonic mean of precision and recall, providing a balanced metric that accounts for both false positives and false negatives:
  (15) $$F1-\mathrm{score}=2\cdot \frac{\mathrm{Precision}\cdot \mathrm{Recall}}{\mathrm{Precision}+\mathrm{Recall}}$$
- **Specificity**: Indicates the proportion of true negatives correctly identified among all actual negative cases. This metric is crucial in medical diagnostics where reducing false alarms is important:
  (16) $$\mathrm{Specificity}=\frac{TN}{TN+FP}$$
- **95% Confidence Interval (CI)**: Represents the range within which the true value of the performance metric is expected to lie with 95% certainty. It accounts for variability across cross-validation folds and quantifies the robustness of the model.
- ***p*****-value**: Used to assess the statistical significance of performance differences between models. A low *p*-value (typically <0.05) suggests that the observed performance difference is unlikely due to chance and, therefore, statistically significant.

These metrics, when analyzed together, provide a holistic view of each model’s diagnostic capability. Especially in the context of medical imaging, high sensitivity ensures that pathological cases are not missed, while high specificity avoids unnecessary misdiagnosis. Furthermore, statistical metrics like confidence intervals and *p*-values reinforce the reliability of the reported results.

### 3.3. Feature Extraction Using Multifractal Analysis

Multifractal analysis was performed to capture the geometric complexity and textural variations of retinal images. This method provides comprehensive quantification of local singularities and global structures in images, making it particularly suitable for detecting pathological changes such as diabetic retinopathy (DR).

From each image, a total of nine multifractal features were extracted: generalized fractal dimensions ($D_{b}$, $D_{i}$, $D_{c}$), singularity spectrum features $\alpha_{min}$, $\alpha_{max}$, $\alpha_{center}$, $f{\left(\alpha \right)}_{max}$), and derived metrics ($spectrum_{w}idth$, symmetric_shift). These features serve as compact and informative descriptors for subsequent classification tasks.

Table 4 presents the extracted features for a sample of 10 images, including both normal and DR cases, to illustrate the variation across different classes.

#### Statistical Summary of Multifractal Features

The overall understanding of the multifractal features extracted from the image dataset was obtained through statistical analysis performed on all samples. Table 5 presents descriptive statistics (count, mean, standard deviation, minimum, quartiles, and maximum) for each multifractal feature, including the box-counting dimension ($D_{b}$), information dimension ($D_{i}$), correlation dimension ($D_{c}$), spectrum width, symmetric shift, minimum Hölder exponent ($\alpha_{\min}$), maximum Hölder exponent ($\alpha_{\max}$), central Hölder exponent ($\alpha_{\mathrm{center}}$), and the maximum of the multifractal spectrum ($f{\left(\alpha \right)}_{\max}$).

**Figure 5:**
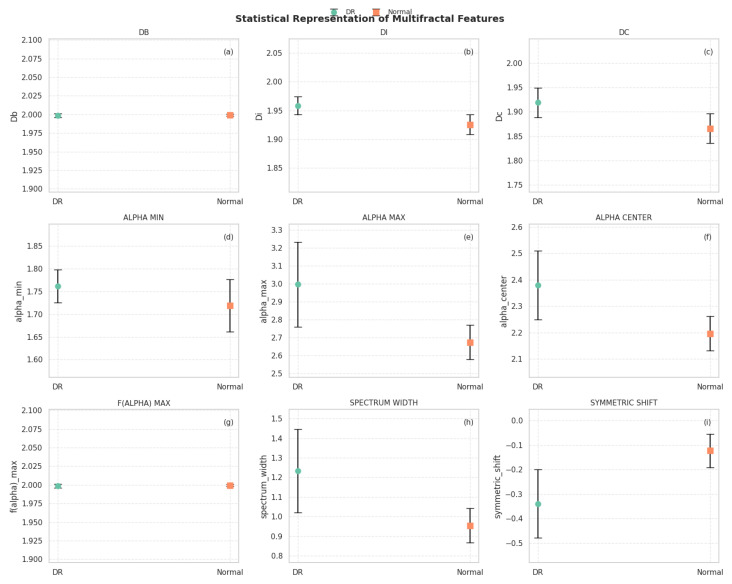
Statistical representation of multifractal features between degraded (DR) and normal regions. (**a**) DB; (**b**) DI; (**c**) DC; (**d**) ALPHA MIN; (**e**) ALPHA MAX; (**f**) ALPHA CENTER; (**g**) F(ALPHA) MAX; (**h**) SPECTRUM WIDTH; (**i**) SYMMETRIC SHIFT.

The feature $D_{b}$ shows very low variance, with values consistently close to 2, highlighting a high level of structural complexity across the dataset. In contrast, the multifractal spectrum parameters $\alpha_{\min}$, $\alpha_{\max}$, and spectrum width exhibit broader dispersion, reflecting greater sensitivity to textural variations. The symmetric shift metric spans both negative and positive values, indicating a slight asymmetry in the multifractal spectra of the images. In addition to the global summary, Figure 5 illustrates the comparative distributions of the multifractal features between two sample categories: degraded regions (DRs) and normal regions. Each subplot reports the mean and standard deviation of the values for one descriptor, enabling a direct visual comparison.

From this visualization, several observations can be highlighted:

- The box-counting dimension ($D_{b}$) and $f{\left(\alpha \right)}_{\max}$ show very low variability and nearly identical mean values across both groups, confirming their global stability.
- Slightly higher values for $D_{i}$ and $D_{c}$ are observed in DR regions, suggesting increased complexity and heterogeneity in those areas.
- The descriptors $\alpha_{\max}$ and $\alpha_{\mathrm{center}}$ are notably higher in DR samples, while $\alpha_{\min}$ remains relatively stable, indicating broader multifractal spectra in degraded regions.
- The spectrum width is significantly greater in DR areas, reflecting stronger variability in singularities and supporting the hypothesis of increased structural irregularity.
- The symmetric shift presents more negative values in DR regions, implying asymmetry in the multifractal spectrum skewed toward higher singularities, which may correspond to abrupt spectral transitions due to vegetation degradation.

These results suggest that multifractal descriptors can capture subtle yet meaningful variations in hyperspectral texture, particularly useful in distinguishing between healthy and degraded vegetation. These features are thus expected to provide valuable input for the subsequent classification process.

### 3.4. Classification Using Machine Learning Algorithms

To identify the most suitable classification strategy for our OCT-based multifractal feature set, we conducted a comparative evaluation of several conventional machine learning classifiers, including Support Vector Machine (SVM), k-nearest neighbors (k-NN), Decision Tree (DT), and Multi-Layer Perceptron (MLP). The choice of these models was motivated by the moderate size and low dimensionality of the extracted feature space, which comprised 9 multifractal descriptors per image. Deep learning approaches, such as convolutional neural networks (CNNs), were deliberately excluded due to their high data requirements and reliance on raw image inputs. In contrast, our handcrafted feature-based pipeline provides interpretable descriptors and enables efficient training with limited computational resources. This strategy ensures clinical scalability and maintains interpretability, which is essential for translational deployment in real-world ophthalmic workflows.

A comprehensive comparison of conventional and advanced machine learning classifiers was carried out to assess the discriminative power of multifractal descriptors extracted from OCT images. A 5-fold cross-validation procedure was employed to ensure robustness of evaluation and reduce variance due to data partitioning. The classification performance of each model was evaluated based on four key metrics: accuracy, precision, recall, and F1-score. Table 6 summarizes the detailed results obtained across the five validation folds for each classifier.

The results presented in Table 6 highlight several important observations. Firstly, all tested models significantly outperform the baseline random chance level (p<0.05), confirming that the multifractal descriptors contain relevant information for distinguishing between normal and non-proliferative diabetic retinopathy (NPDR) cases.

Among classical classifiers, the Support Vector Machine (SVM) and Decision Tree (DT) exhibit promising results, with average accuracies of 91.52% and 91.92%, respectively. These results underline the capability of basic discriminative and rule-based models to capture some degree of structure in the multifractal feature space.

Ensemble-based methods such as Random Forest (RF) and Gradient Boosting (GB) yield improved results, achieving average accuracies around 94.45% and 94.97%, respectively. These techniques are known for their robustness to overfitting and their ability to aggregate multiple weak learners, which explains their enhanced performance.

The most notable improvements come from state-of-the-art Gradient Boosting frameworks—XGBoost and LightGBM—which achieve accuracies of 95.27% and 94.97%, respectively. These models are particularly efficient in handling high-dimensional data and are optimized for speed and accuracy, making them suitable for complex medical classification tasks.

The Multi-Layer Perceptron (MLP), a type of feedforward artificial neural network, achieves the highest performance across all metrics, with an average accuracy of 98.02% and an F1-score of 98.01%. The superior results of the MLP suggest that neural networks are particularly adept at learning the complex, nonlinear relationships embedded in the multifractal descriptors. This underscores the relevance of deep learning architectures in medical image analysis and strengthens the case for their use in automated DR screening systems. While classical machine learning algorithms provide a solid baseline for classification, the use of advanced ensemble techniques and neural networks leads to significant improvements in detection accuracy. These findings support the feasibility of leveraging multifractal analysis combined with powerful classifiers such as MLP for the accurate diagnosis of NPDR.

**Figure 6:**
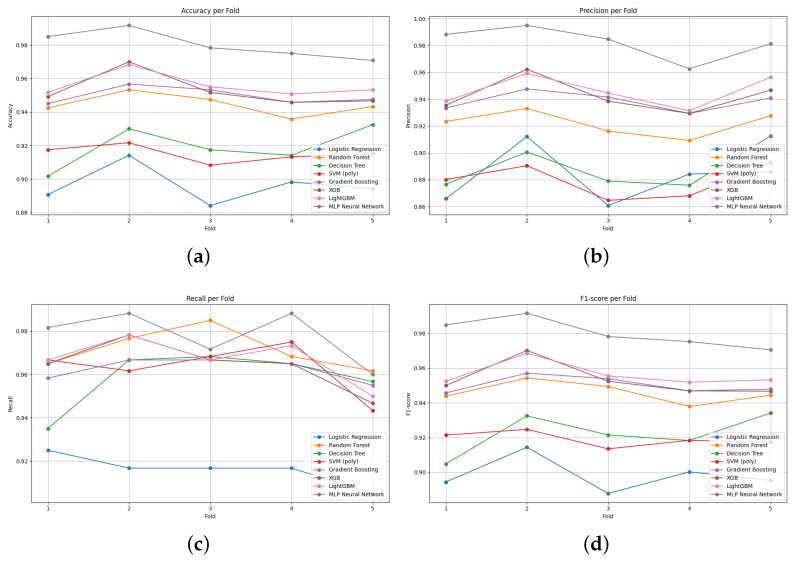
Performance variation of classification models across 5-fold cross-validation: (**a**) accuracy, (**b**) precision, (**c**) recall, and (**d**) F1-score. Each curve represents the performance of one classifier evaluated on multifractal features.

Figure 6 illustrates the comparative performance of various classical and ensemble-based classifiers across five folds of cross-validation using four evaluation metrics: accuracy, precision, recall, and F1-score.

From Figure 6a it is evident that the **MLP Neural Network** and **LightGBM** consistently outperformed other models in terms of accuracy, maintaining values close to 0.96 and 0.95, respectively. Conversely, traditional classifiers such as **Logistic Regression** and **Decision Tree** exhibited the lowest accuracy scores, suggesting limited capacity to capture the complexity of the multifractal features.

Figure 6b highlights the performance in terms of precision. Again, the **MLP Neural Network** achieved near-perfect precision values across all folds, indicating its strong ability to avoid false positives. Other ensemble models like **XGBoost** and **LightGBM** also demonstrated relatively high and stable precision across folds, reinforcing their robustness.

In Figure 6c which presents recall, the results show that **Random Forest** slightly outperformed other models in some folds. However, the MLP and ensemble-based models (LightGBM, XGBoost, Gradient Boosting) generally maintained higher recall compared to traditional classifiers, indicating superior sensitivity in identifying true positives.

Figure 6d summarizes the balanced performance via the F1-score. The **MLP Neural Network** consistently attained the highest F1-scores, followed by **LightGBM**, showcasing their ability to maintain a good trade-off between precision and recall. In contrast, Logistic Regression and Decision Tree were less effective, displaying notable fluctuations and lower mean F1-scores.

In summary, the results suggest that while classical models provide a basic level of performance, ensemble learning techniques and neural network-based models significantly enhance classification performance when applied to multifractal features extracted from retinal images. The MLP Neural Network, in particular, stands out as the most reliable classifier in terms of all four metrics.

**Figure 7:**
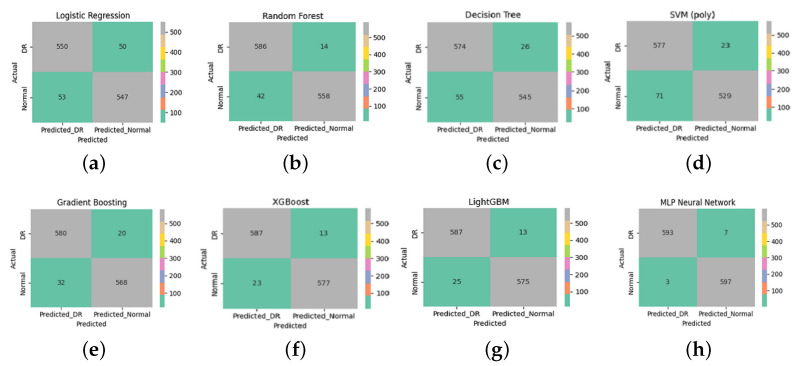
Confusion matrices for the evaluated supervised learning models. Each subfigure corresponds to (**a**) Logistic Regression, (**b**) Random Forest, (**c**) Decision Tree, (**d**) SVM (polynomial), (**e**) Gradient Boosting, (**f**) XGBoost, (**g**) LightGBM, and (**h**) MLP Neural Network. These matrices illustrate the classification performance for diabetic retinopathy (DR) and normal cases using features extracted via multifractal analysis.

Figure 7 presents the confusion matrices for eight supervised learning models applied to the classification of diabetic retinopathy (DR) and normal retinal images. These matrices detail the counts of true positives (correctly identified DR), true negatives (correctly identified normal cases), false positives, and false negatives.

The MLP Neural Network (Figure 7h) achieved the best classification results, with only seven DR cases misclassified as normal (false negatives) and three normal cases misclassified as DR (false positives). This indicates high accuracy, sensitivity, and specificity, reflecting the model’s capacity to generalize well when fed with discriminative features.

A qualitative analysis of the misclassified images revealed that false negatives often corresponded to OCT scans with minimal or subtle structural disruption, potentially below the detection threshold of multifractal descriptors. Conversely, the few false positives were frequently associated with normal images showing noise-like or irregular texture patterns that resembled early pathological changes, leading to over-sensitive detection.

Both XGBoost (Figure 7f) and LightGBM (Figure 7g) also demonstrated strong classification performance, with minimal misclassification (13 false negatives and 23–25 false positives). These gradient-boosting algorithms benefit from their ensemble nature, enabling them to capture complex patterns and interactions within the multifractal features.

The Random Forest model (Figure 7b) also performed well, with 14 false negatives and 42 false positives, outperforming the Decision Tree (Figure 7c), which showed higher misclassification rates. This highlights the benefit of ensemble methods in reducing overfitting and variance compared to a single tree classifier.

Although the SVM with a polynomial kernel (Figure 7d) achieved reasonable results, it exhibited a relatively higher number of false positives (71). It is important to note, however, that this behavior is not due to poor parameter tuning—since the model was tested with the best-performing configuration after thorough optimization—but rather suggests a limitation of this particular kernel type in separating.

Logistic Regression (Figure 7a) achieved balanced but modest results, with 50 false negatives and 53 false positives. This shows its limited ability to capture the data’s nonlinear structure despite the use of advanced features.

From a clinical standpoint, minimizing false negatives is crucial to prevent missing early-stage diabetic retinopathy (DR) cases, while false positives may lead to unnecessary referrals or patient anxiety. Despite the strong overall performance, the few remaining misclassified cases are likely attributable to ultra-early DR manifestations, where structural changes are minimal or subclinical. These borderline cases highlight the limitations of relying solely on OCT-based structural descriptors and underscore the potential benefit of integrating complementary functional modalities. As a future direction, combining our multifractal OCT approach with functional biomarkers such as Electroretinography (ERG) could enhance sensitivity to early neuroretinal dysfunction and reduce diagnostic uncertainty in ambiguous presentations. Overall, these findings emphasize the importance of using robust and expressive classifiers in conjunction with informative feature extraction techniques. In this study, multifractal analysis played a crucial role in characterizing the spatial and textural complexity of retinal images. By capturing multi-scale structural variations, this method provided a rich set of discriminative features that significantly enhanced classification performance across all models.

The consistently high accuracy obtained, particularly by ensemble and deep learning models, underlines the effectiveness of the multifractal analysis in preprocessing and transforming retinal data into a more learnable representation. This reinforces its necessity as a key step in the diagnostic pipeline, enabling better generalization and more reliable detection of diabetic retinopathy.

Table 7 presents the performance metrics obtained for each classification model, including accuracy, precision, sensitivity (recall), specificity, F1-score, and the 95% confidence interval for the accuracy.

The results in Table 7 highlight the excellent discriminative power achieved by all classifiers. Among them, the MLP Neural Network attained the highest overall accuracy (98.02%), demonstrating its superior ability to learn complex nonlinear representations from the multifractal features extracted. It also exhibited excellent precision (98.24%), sensitivity (97.80%), and specificity (98.84%), though the confidence interval was not reported due to deterministic results on cross-validation folds. Tree-based ensemble methods also performed very competitively:

- LightGBM achieved an accuracy of 95.58% with a narrow confidence interval [94.42%–96.74%], indicating a robust and reliable performance.
- XGBoost and Gradient Boosting followed closely, with accuracies of 95.27% and 94.97%, respectively.

Traditional classifiers such as Random Forest (94.45%) and Logistic Regression (89.63%) also delivered good results, but with slightly lower precision and sensitivity compared to boosting methods and the neural network. The Decision Tree and SVM with polynomial kernel achieved accuracies above 91%, but they exhibited wider confidence intervals, suggesting less stability across different folds.

Overall, these findings confirm that multifractal-based feature extraction, combined with modern machine learning models, can achieve highly accurate and reliable diabetic retinopathy detection from OCT images. The use of ensemble learning and deep architectures provided notable improvements compared to simpler models, particularly in terms of generalization and sensitivity to pathological variations.

The experimental findings demonstrate the strong discriminative ability of multifractal features extracted from OCT images for the early detection of diabetic retinopathy. Among the various classifiers evaluated, the MLP Neural Network consistently outperformed other models across all evaluation metrics, highlighting the effectiveness of combining multifractal analysis with deep learning techniques in modeling the complex structural alterations associated with early retinal pathology.

Ensemble-based methods, such as LightGBM and XGBoost, also exhibited excellent performance, further validating the suitability of gradient-boosting strategies when working with multifractal descriptors. Overall, the proposed pipeline underscores the robustness, scalability, and practical applicability of multifractal analysis on OCT images. Unlike previous studies that primarily utilized OCTA datasets, this work demonstrates that standard OCT imaging—when combined with advanced mathematical modeling—can serve as an equally powerful and more accessible tool for early diabetic retinopathy screening.

### 3.5. Performance Comparison with State-of-the-Art Methods

To evaluate the effectiveness of the proposed multifractal-based MLP classification approach, we conducted a comparative analysis with several state-of-the-art machine learning and deep learning methods previously applied to retinal image classification. Table 8 summarizes the key performance metrics—accuracy, sensitivity, specificity—as well as dataset characteristics and imaging modalities used in these studies.

As shown in Table 8, our proposed method achieves competitive or superior performance compared to recent approaches, with 98.02% accuracy, 97.80% sensitivity, and 98.84% specificity on 6000 OCT images. While some methods demonstrate marginally higher sensitivity (e.g., SVM + GLCM [41]), they often underperform in terms of overall accuracy or specificity. Additionally, approaches relying on OCTA [12] or massive annotated datasets [39] may not be feasible in routine clinical settings.

In contrast, our model operates on standard OCT images and uses mathematically grounded multifractal features, ensuring both performance and interpretability. This makes it especially suited for integration into real-world ophthalmic workflows, including in primary care or resource-limited settings where early intervention is critical. Unlike deep learning models that often act as “black boxes,” the proposed method provides transparent structural descriptors that facilitate clinical validation and enhance trust among ophthalmologists.

Moreover, the model’s low computational requirements enable deployment on existing diagnostic platforms without the need for specialized hardware or extensive data labeling. By detecting subtle structural changes in retinal layers—often invisible in fundus images—our framework supports truly early-stage diabetic retinopathy screening. Future development will focus on an integrated, user-friendly interface and explainable AI tools to visually highlight pathologically relevant regions, further bridging the gap between algorithmic decision-making and clinical practice.

While the present study establishes that multifractal features extracted from OCT images offer a robust structural biomarker framework for early diabetic retinopathy (DR) detection, future work should advance toward integrating complementary functional modalities to achieve a more comprehensive and biologically faithful diagnostic model. Optical Coherence Tomography (OCT) remains an indispensable structural imaging technique, providing high-resolution visualization of retinal microarchitecture, including the inner retinal layers and retinal pigment epithelium. However, as a purely anatomical modality, OCT cannot interrogate the functional state of retinal neurons, which may be compromised in the earliest neurodegenerative stages of DR, well before visible vascular or tissue disruption occurs. In contrast, Electroretinography (ERG) offers objective, layer-specific assessment of retinal function by recording bioelectrical responses to photic stimuli. Notably, recent studies have demonstrated that ERG abnormalities—particularly reduced oscillatory potentials and attenuated photopic negative response (PhNR)—can be detected in diabetic patients, thus revealing latent inner retinal dysfunction [43,44]. This structural–functional dissociation highlights a critical limitation of relying solely on OCT-based morphological features and underscores the need for multimodal approaches that capture both tissue integrity and neurophysiological performance. Future research should explore the fusion of OCT-derived multifractal descriptors with ERG-derived electrophysiological biomarkers within integrated machine learning pipelines. Such hybrid models may yield more sensitive and specific detection of subclinical DR, enabling earlier therapeutic intervention. Moreover, this multimodal paradigm aligns with the goals of precision ophthalmology, offering a path toward personalized screening, disease staging, and longitudinal monitoring in diabetic eye care.

## 4. Conclusions

The results of this study confirmed the high effectiveness of combining multifractal analysis with machine learning classifiers for the early detection of diabetic retinopathy using OCT images. Our proposed framework successfully captured subtle structural irregularities associated with early retinal degeneration, achieving highly accurate and reliable classification performances, particularly with the MLP Neural Network.

This work’s key contribution lies in demonstrating that standard OCT imaging when coupled with multifractal mathematical modeling, can provide a powerful, accessible, and cost-effective alternative to more complex imaging techniques like OCTA. This highlights the proposed method’s clinical applicability and scalability for real-world screening programs and ophthalmologic diagnosis.

For future work, we aim to extend the proposed framework to detect and classify other retinal diseases, such as age-related macular degeneration, glaucoma, etc., thus validating its generalizability across diverse pathological conditions. Although this study focused on the binary classification between normal and diabetic retinopathy (DR) images, we acknowledge that specific OCT manifestations of DR may partially resemble those of other ocular pathologies such as retinal vein occlusion (RVO) and choroidal neovascularization (CNV). Our dataset already includes such retinal conditions, and future extensions of this work will address multi-class classification settings to enhance the specificity and reduce potential diagnostic ambiguities. We also plan to evaluate the method on larger and multi-class datasets and explore its adaptability to other imaging modalities. Additionally, future work will involve validating the proposed approach on external OCT datasets from different clinical centers to assess its robustness and generalizability across varied acquisition conditions. Finally, efforts will focus on optimizing the system for real-time implementation to facilitate its integration into clinical practice.

## Figures and Tables

**Table 1:** Summary of extracted multifractal features and their clinical relevance in diabetic retinopathy detection.

Feature	Clinical Significance for DR Detection
$D_{B}$	Global complexity—detects microstructural degradation
$D_{I}$	Heterogeneity—captures irregularity from lesions
$D_{C}$	Local clustering—identifies lesion aggregation
$\alpha_{\min}$	Highlights sharp transitions due to hemorrhages
$\alpha_{\max}$	Indicates smooth healthy tissue
$\alpha_{\mathrm{center}}$	Describes dominant texture regularity
$f{\left(\alpha \right)}_{\max}$	Abundance of dominant structural type
Spectrum Width	Degree of pathological texture variability
Symmetric Shift	Skewness toward coarse or fine pathological patterns

**Table 2:** Comparison of multifractal features between the DR case (DR_141) and healthy control (Normal_141).

Feature	DR_141	Normal_141
$D_{B}$	1.9994	1.9999
$D_{I}$	1.9418	1.9233
$D_{C}$	1.8911	1.8492
$\alpha_{\min}$	1.7580	1.6238
$\alpha_{\max}$	3.0848	2.6137
$\alpha_{\mathrm{center}}$	2.4214	2.1188
$f{\left(\alpha \right)}_{\max}$	1.9994	1.9999
Spectrum Width Δα	1.3269	0.9900
Symmetry Shift	−0.369	−0.048

**Table 3:** Optimized hyperparameters and final configurations for all classifiers.

Classifier	Hyperparameter	Optimized Value
Logistic Regression	Solver	liblinear
	Penalty	L2
	C (Regularization)	1.0
Random Forest	Number of Estimators	100
	Maximum Depth	10
Decision Tree	Maximum Depth	5
	Criterion	entropy
SVM (Poly Kernel)	Kernel	poly
	Degree	3
	C	1.0
	Probability Estimation	True
Gradient Boosting	Number of Estimators	100
	Learning Rate	0.1
	Maximum Depth	3
XGBoost	Number of Estimators	100
	Learning Rate	0.1
	Maximum Depth	4
	Eval Metric	mlogloss
LightGBM	Number of Estimators	100
	Learning Rate	0.1
	Maximum Depth	5
MLP Neural Network	Hidden Layers	(128, 64)
	Activation Function	ReLU
	Solver	Adam
	Max Iteration	1000
	Random State	42

**Table 4:** Sample of 10 retinal images with extracted multifractal features.

ID	$D_{b}$	$D_{i}$	$D_{c}$	$\alpha_{\min}$	$\alpha_{\max}$	$\alpha_{\mathrm{center}}$	$f{\left(\alpha \right)}_{\max}$	Width	Shift	Class
**2518**	1.9861	1.9741	1.9577	1.8185	3.1086	2.4635	1.9861	1.2901	−0.4656	DR
**148**	1.9986	1.9751	1.9489	1.7844	3.3101	2.5472	1.9986	1.5257	−0.5244	DR
**465**	1.9993	1.9265	1.8636	1.7101	2.6867	2.1984	1.9993	0.9766	−0.1289	Normal
**981**	1.9996	1.9365	1.8869	1.7676	3.0968	2.4322	1.9996	1.3292	−0.3706	DR
**2518**	2.0000	1.8965	1.8064	1.6961	2.6377	2.1669	2.0000	0.9415	−0.0778	Normal
**600**	2.0000	1.9397	1.8934	1.7486	2.5678	2.1582	2.0000	0.8192	−0.0955	Normal
**2489**	1.9985	1.9755	1.9548	1.7930	3.0982	2.4456	1.9985	1.3053	−0.4097	DR
**2809**	1.9980	1.9432	1.8961	1.7445	2.7306	2.2376	1.9980	0.9861	−0.1810	Normal
**2951**	2.0000	1.9202	1.8480	1.6436	2.5938	2.1187	2.0000	0.9502	−0.0446	Normal
**1166**	1.9933	1.9707	1.9532	1.8027	3.2319	2.5173	1.9933	1.4291	−0.4814	DR

**Table 5:** Statistical summary of multifractal features (6000 samples).

Feature	Mean	Std	Min	25%	Median	Max
$D_{b}$	1.9990	0.0022	1.9709	1.9989	1.9999	2.0000
$D_{i}$	1.9419	0.0233	1.8467	1.9264	1.9413	1.9873
$D_{c}$	1.8925	0.0402	1.7425	1.8665	1.8900	1.9758
$\alpha_{\min}$	1.7404	0.0528	1.4852	1.7096	1.7396	1.9599
$\alpha_{\max}$	2.8345	0.2417	2.2947	2.6331	2.7528	3.3733
$\alpha_{\mathrm{center}}$	2.2874	0.1380	2.0051	2.1727	2.2485	2.5845
$f{\left(\alpha \right)}_{\max}$	1.9990	0.0022	1.9709	1.9989	1.9999	2.0000
Spectrum width	1.0941	0.2150	0.5393	0.9217	1.0229	1.5837
Symmetric shift	−0.2312	0.1540	−0.5736	−0.3686	−0.1830	0.0722

**Table 6:** Performance comparison of various classifiers over 5-fold cross-validation using multifractal features. Metrics reported include accuracy, precision, recall, and F1-score.

Model	Fold	Accuracy	Precision	Recall	F1-Score
LogisticRegression	1	0.8908	0.8658	0.9250	0.8944
	2	0.9142	0.9121	0.9167	0.9144
	3	0.8842	0.8607	0.9167	0.8878
	4	0.8983	0.8842	0.9167	0.9002
	5	0.8942	0.8858	0.9050	0.8953
	**Average**	**0.8963**	**0.8817**	**0.9160**	**0.8984**
	**IC 95% (Acc.)**	**0.8791–0.9136**			
	***p*** **-value (vs. 0.50)**	p<0.05			
Random Forest	1	0.9425	0.9234	0.9650	0.9438
	2	0.9533	0.9331	0.9767	0.9544
	3	0.9475	0.9163	0.9850	0.9494
	4	0.9358	0.9092	0.9683	0.9379
	5	0.9433	0.9277	0.9617	0.9444
	**Average**	**0.9445**	**0.9219**	**0.9714**	**0.9460**
	**IC 95% (Acc.)**	**0.9316–0.9574**			
	***p*** **-value (vs. 0.50)**	p<0.05			
Decision Tree	1	0.9017	0.8766	0.9350	0.9048
	2	0.9300	0.9006	0.9667	0.9325
	3	0.9175	0.8790	0.9683	0.9215
	4	0.9142	0.8759	0.9650	0.9183
	5	0.9325	0.9126	0.9567	0.9341
	**Average**	**0.9192**	**0.8889**	**0.9583**	**0.9222**
	**IC 95% (Acc.)**	**0.9038–0.9346**			
	***p*** **-value (vs. 0.50)**	p<0.05			
SVM	1	0.9175	0.8801	0.9667	0.9214
	2	0.9217	0.8904	0.9617	0.9247
	3	0.9083	0.8646	0.9683	0.9135
	4	0.9133	0.8680	0.9750	0.9184
	5	0.9150	0.8927	0.9433	0.9173
	**Average**	**0.9152**	**0.8792**	**0.9630**	**0.9191**
	**IC 95% (Acc.)**	**0.8994–0.9309**			
	***p*** **-value (vs. 0.50)**	p<0.05			
Gradient Boosting	1	0.9450	0.9334	0.9583	0.9457
	2	0.9567	0.9477	0.9667	0.9571
	3	0.9533	0.9416	0.9667	0.9539
	4	0.9458	0.9294	0.9650	0.9469
	5	0.9475	0.9409	0.9550	0.9479
	**Average**	**0.9497**	**0.9386**	**0.9623**	**0.9503**
	**IC 95% (Acc.)**	**0.9373–0.9620**			
	***p*** **-value (vs. 0.50)**	p<0.05			
XGBoost	1	0.9492	0.9354	0.9650	0.9500
	2	0.9700	0.9623	0.9783	0.9702
	3	0.9517	0.9385	0.9667	0.9524
	4	0.9458	0.9294	0.9650	0.9469
	5	0.9467	0.9467	0.9467	0.9467
	**Average**	**0.9527**	**0.9425**	**0.9643**	**0.9532**
	**IC 95% (Acc.)**	**0.9407–0.9646**			
	***p*** **-value (vs. 0.50)**	p<0.05			
LightGBM	1	0.9517	0.9385	0.9667	0.9524
	2	0.9683	0.9592	0.9783	0.9686
	3	0.9550	0.9446	0.9667	0.9555
	4	0.9508	0.9314	0.9733	0.9519
	5	0.9533	0.9564	0.9500	0.9532
	**Average**	**0.9497**	**0.9460**	**0.9670**	**0.9563**
	**IC 95% (Acc.)**	**0.9373–0.9620**			
	***p*** **-value (vs. 0.50)**	p<0.05			
MLP	1	0.9850	0.9883	0.9817	0.9849
	2	0.9917	0.9950	0.9883	0.9916
	3	0.9783	0.9848	0.9717	0.9782
	4	0.9750	0.9627	0.9883	0.9753
	5	0.9708	0.9813	0.9600	0.9705
	**Average**	**0.9802**	**0.9824**	**0.9780**	**0.9801**
	**IC 95% (Acc.)**	**0.9724–0.9879**			
	***p*** **-value (vs. 0.50)**	p<0.05			

**Table 7:** Performance metrics of different classification algorithms on OCT image classification.

Algorithm	Accuracy	Precision	Sensitivity	Specificity	F1-Score	95% CI (Accuracy)
Logistic Regression	0.8963	0.8817	0.9160	0.9162	0.8984	[0.8791–0.9136]
Random Forest	0.9445	0.9219	0.9713	0.9755	0.9460	[0.9316–0.9574]
Decision Tree	0.9192	0.8889	0.9583	0.9545	0.9222	[0.9038–0.9346]
SVM (poly)	0.9152	0.8792	0.9630	0.9583	0.9191	[0.8994–0.9309]
Gradient Boosting	0.9497	0.9386	0.9623	0.9660	0.9503	[0.9373–0.9620]
XGBoost	0.9527	0.9424	0.9643	0.9780	0.9532	[0.9407–0.9646]
LightGBM	0.9558	0.9460	0.9670	0.9779	0.9563	[0.9442–0.9674]
MLP Neural Network	0.9802	0.9824	0.9780	0.9884	0.9801	[0.9724–0.9879]

**Table 8:** Performance comparison with state-of-the-art methods.

Methods	Accuracy	Sensitivity	Specificity	Samples	Type of Images
CNN	97%	94%	98%	400	Fundus
KNN+ Fractal analysis [38]	89.17%	-	-	120	Fundus
Entropy CNN [39]	86.1%	73.24%	93.81%	21,123	Fundus
DNN + PCA [40]	97%	92%	95%	1151	OCTA
(SVM) + GLCM [41]	82%	98%	89%	-	Fundus
CNN + SVM [42]	94%	100%	88%	104	OCT
Multifractal + SVM [12]	98.5%	100%	97.3%	170	OCTA
Multifractal + MLP (our study)	**98.02%**	**97.80%**	**98.84%**	**6000**	**OCT**

## Data Availability

The data presented in this study are openly available in: https://www.kaggle.com/datasets/obulisainaren/retinal-oct-c8 (accessed on 4 January 2025).
